# Generative AI in aging societies: the risk of a double divide

**DOI:** 10.3389/frai.2026.1973907

**Published:** 2026-10-14

**Authors:** Alexander Seifert

**Affiliations:** School of Social Work, Institute for Integration and Participation, University of Applied Sciences and Arts Northwestern Switzerland, Olten, Switzerland

**Keywords:** age-inclusive AI, ageism, AI literacy, algorithmic bias, digital inequality, generative artificial intelligence, older adults, representational harm

## 1 Introduction

Population aging and the rapid diffusion of generative artificial intelligence are two defining transformations of the twenty-first century, yet they are rarely considered together. Generative artificial intelligence (generative AI) is moving from a technological novelty to an everyday infrastructure. It drafts emails, summarizes documents, produces images, answers health questions and increasingly mediates first-line interactions with organizations. Public debate has focused heavily on schools, universities, workplaces, productivity, misinformation and regulation. Much less attention has been paid to how this transformation intersects with population aging and the increasingly digital organization of later life. This omission matters because generative AI is not simply another digital tool. It is becoming part of the infrastructure through which people search for knowledge, assess risks, access services and imagine social groups.

In this article, ‘older adults' refers broadly to people in later life. The term accommodates differences in age definitions across national and research contexts. Older adults are not a marginal user group; they are a growing share of the population in many countries and are increasingly expected to navigate digital welfare, health, financial and communication systems. Yet research and design practices often continue to position older adults as late adopters, vulnerable users or recipients of technological solutions rather than as citizens whose agency, knowledge and social representation matter in the future AI ecosystem (Mannheim et al., 2023; Choi et al., 2026).

Existing concepts provide distinct perspectives on the problem at hand. Research on digital inequality has established the “digital divide” as a multidimensional phenomenon, spanning disparities in access, digital skills, patterns of use, and resultant outcomes (Dijk, 2020; Bentley et al., 2024). Complementarily, research on digital ageism investigates age-based assumptions embedded in digital technologies, including how these assumptions generate and sustain inequalities in both access and participation, as well as how ageism and patterns of digital engagement mutually shape one another (Mannheim and Köttl, 2024).

Building on these literatures, the proposed framework offers a targeted synthesis for generative AI. Specifically, it links older adults' opportunities to use and to shape generative AI systems with the ways aging is represented in AI-generated text and images. The framework advances the claim that when older adults' perspectives are insufficiently included during the design and evaluation of generative AI, stereotypical representations may remain effectively unchallenged. In turn, these representations may undermine older adults' willingness to engage with such systems.

The proposed double-divide framework distinguishes inequality in engaging with generative AI (Wang et al., 2025) from inequality in being represented by it (Chu et al., 2022): first, older adults may face an *AI Participation Divide*: unequal opportunities to access and use generative AI and to interpret and critically evaluate its outputs. Second, they may face an *AI Representation Divide*: inequalities in how aging and older adults are represented through AI-generated text, images and decisions. These two divides are distinct but connected. Together, they may shape who benefits from AI and how aging itself is understood.

## 2 The promise of generative AI for older adults

Generative AI can offer substantial benefits in later life. Its natural language interface may lower the barriers that made earlier digital technologies difficult to use. A person who struggles with search engines, online forms or administrative language may ask a chatbot to explain a letter, draft a response, translate information or compare options. Such functions could support autonomy and participation when they are reliable, accessible and embedded in meaningful social contexts. Unlike many earlier digital technologies, generative AI does not require users to navigate complex menus or predefined categories. Its conversational form may allow older adults to start with questions in their own words. In health, AI-driven tools may help explain medical information, support appointment preparation, make complex instructions easier to understand and offer new forms of communication support. For older adults with sensory limitations, low digital confidence or limited formal education, conversational interfaces could be more approachable than menu-based systems. Emerging work on AI-based health technologies and assistive technologies suggests that older adults often see potential benefits, especially when technologies are useful, understandable, trustworthy and aligned with their everyday lives (Enam et al., 2025; Wong et al., 2025). This promise matters. If generative AI is well designed, it could become an important tool for inclusion. The problem is that benefits do not flow automatically from availability. Access to an AI system does not mean that people know what to ask, how to interpret answers or how to judge the accuracy of the output. This is where the first divide begins.

## 3 Risk one: the AI participation divide

In the age of generative AI, the crucial question is not only whether older adults can access generative AI but also whether they have the competence to use it effectively and critically. The following four layers describe the participation divide.

The first layer is access and use: generative AI may be freely available through search engines, smartphones and apps, but this does not mean that its use is evenly distributed. Evidence on AI-related competencies shows that vulnerable user groups with lower AI knowledge and skills are more likely to be older, less educated and less equipped with privacy-protection skills (Wang et al., 2025). Behavioral evidence on ChatGPT use likewise points to generational and educational differences in adoption (Kacperski et al., 2025). These findings suggest that AI may extend rather than erase established inequalities in digital participation.

The second layer is meaningful use: generative AI requires a new repertoire of practical skills. Users need to know what AI can do, which tasks it is appropriate for, how to formulate prompts, when to provide context and when not to disclose sensitive information. These are not trivial skills. They are learned through experimentation, social support and prior digital confidence. Existing digital experiences can therefore spill over into relationships with AI: people who have felt excluded from digital technology may approach AI with lower confidence, fewer positive experiences and less trust (Bentley et al., 2024).

The third layer is interpretation: AI outputs often sound fluent, confident and authoritative, even when they are incomplete, biased or wrong. Older adults, like all users, need to understand that generative AI produces plausible responses rather than verified truth. This distinction is especially important when AI is used for health information, financial questions or legal guidance. The danger is not only non-use but overtrust: a person may use AI and still be disadvantaged because they cannot recognize the limits of the answer.

The fourth layer is evaluation. This may become the defining competence of the generative AI era. Evaluation competence includes more than checking whether a statement is factually correct. It also involves recognizing when an image, voice, recommendation or personalized message may have been synthetically generated, manipulated or designed to exploit trust. For older adults, this is particularly crucial in domains where trust relationships matter, such as health advice, social communication, banking and public services. Users increasingly need to detect hallucinations, misleading summaries, manipulated images, synthetic voices, scams and AI-generated fake content. Older adults are often framed as vulnerable to online misinformation (Brashier and Schacter, 2020; Li et al., 2025), yet the question should not be reduced to individual deficit. The information environment itself is becoming harder to evaluate, especially for older adults with limited familiarity with generative AI. As services, media and interpersonal communication are increasingly saturated with synthetic content, the critical evaluation of AI becomes a basic condition of social participation. The result is not merely an individual skills gap, but a societal divide in the ability to judge the credibility, authenticity and trustworthiness of AI-mediated information.

## 4 Risk two: the AI representation divide

The second divide concerns representation: the risk that AI-generated portrayals fail to reflect the diversity of later life. The divide lies between the diversity of later life as it is lived and the restricted repertoire through which AI systems make it visible. Generative AI can produce text, images and narratives about aging that circulate through society. When people ask AI systems to describe older adults, design services for aging populations, generate images of later life or summarize what aging means, the outputs participate in shaping cultural understandings of age and may potentially result in ageism (Martens et al., 2025; Osinga et al., 2026). Reviews of digital technology design show that older adults are frequently framed through assumptions of decline, dependency and lack of competence (Mannheim et al., 2023). Work on sociotechnical ageism shows how AI in care and aging contexts can reproduce narrow ideas of old age as a problem to be managed through technical solutions (Neves et al., 2023). A scoping review of age-related bias in AI further shows that age bias may enter AI systems through data, design, modeling and deployment, including through underrepresentation of older adults and biased evaluation of system performance (Chu et al., 2023). Generative AI intensifies this issue because it makes representation scalable. Repetition gives such representations social weight. When similar ageist framings appear across chatbots, image generators, search interfaces and administrative tools, they may come to look less like stereotypes and more like common sense. For example, an analysis of AI images generated at two time points found persistent imbalances in the demographic characteristics depicted (Martens et al., 2025). Such findings concern the content of generated images; they do not establish how widely these images circulate or how exposure affects public attitudes or older adults' self-perceptions. Nevertheless, repeated generation and dissemination of narrow portrayals could reinforce stereotypes about aging. The extent and consequences of this potential reinforcement of stereotypes, however, require empirical investigation across systems, contexts, and audiences.

If AI systems repeatedly associate aging, for example with frailty, loneliness, cognitive decline, burden or dependency, they may normalize a narrow deficit narrative of later life. Later life characterized by competence, political engagement, paid or unpaid work, intimacy, creativity and social contribution can disappear when aging is repeatedly reduced to care needs and decline. Conversely, if diverse forms of aging are underrepresented, the heterogeneous lives of older adults may become invisible. Gallistl et al. (2025) argue that the current relationship between aging and AI should be understood beyond an interventionist logic that treats AI primarily as a solution to the individual problems of older people. Their political economy perspective highlights how AI is embedded in structures of power, markets, policy and material infrastructures that shape the meaning of aging. This perspective draws attention to how AI-generated portrayals might position older adults as recipients of care rather than as people with diverse roles and capabilities.

The representation divide may also reinforce the participation divide. If older adults rarely see themselves represented as competent, diverse and agentic AI users, they may be less likely to identify with AI technologies. If they participate less in AI systems, their preferences, language and experiences may be less visible in feedback loops, training data and design processes. Lower participation can then lead to poorer representation, which in turn can deepen exclusion.

## 5 Discussion

Avoiding a double divide requires more than giving older adults access to AI tools. First, AI literacy initiatives for older adults should account for differences in education, health, socioeconomic circumstances, and previous exposure to technology (Suárez and García-Mariñoso, 2025). Training should build on participants' existing knowledge and offer flexible pacing, accessible materials, and support suited to their needs. Content should include practical prompting, interpretation of uncertainty, privacy protection, and critical evaluation of AI-generated content, including health misinformation, scams, deepfakes, and synthetic media. Second, AI systems should be evaluated for age-related bias not only in technical performance but also in the narratives and images they generate. Third, older adults should be involved as co-designers of AI systems and not merely as test users. Recruitment should reflect the diversity of older adults by including heterogeneous groups of older adults and by addressing power imbalances within the design process. Fourth, essential health, welfare, financial and public services should retain accessible non-AI alternatives. Age-inclusive AI cannot mean making participation conditional on the ability or willingness to interact with automated systems. Fifth, AI governance should treat age as a core dimension of fairness, inclusion and representation. This includes audit practices that examine whether AI systems exclude older adults from benefits or reproduce ageist assumptions in outputs and decisions. The proposed policy and design recommendations are normative proposals informed by the preceding evidence and conceptual analysis. Their effectiveness should be examined empirically across different populations, settings, and generative AI systems.

Age-inclusive AI therefore requires both the capacity to act within AI-mediated environments and the right to be represented by them in ways that reflect the diversity, agency and complexity of later life. Without such efforts, generative AI may widen a double divide in aging societies, one that limits older adults' capacity to act in AI-mediated environments and narrows how aging itself is seen.
